# Descending aortic calcification increases renal dysfunction and in-hospital mortality in cardiac surgery patients with intraaortic balloon pump counterpulsation placed perioperatively: a case control study

**DOI:** 10.1186/cc11162

**Published:** 2012-01-25

**Authors:** Martina Nowak-Machen, James D Rawn, Prem S Shekar, Aya Mitani, Sagun Tuli, Tobias M Bingold, Garrett Lawlor, Holger K Eltzschig, Stanton K Shernan, Peter Rosenberger

**Affiliations:** 1 Department of Anesthesiology, Perioperative and Pain Medicine, Brigham and Women's Hospital, Harvard Medical School, 75 Francis Street, Boston, MA 02115, USA; 2 Department of Cardiac Surgery, Brigham and Women's Hospital, Harvard Medical School, 75 Francis Street, Boston, MA 02115, USA; 3 Medical Statistics, Brigham and Women's Hospital, Harvard Medical School, 75 Francis Street, Boston, MA 02115, USA; 4 Department of Neurosurgery Brigham and Women's Hospital, Harvard Medical School, 75 Francis Street, Boston, MA 02115, USA; 5 Department of Medicine, Beth Israel Deaconess Medical Center, Harvard Medical School, 330 Brookline Avenue, Boston, MA 02115, USA; 6 Department of Anesthesiology, University of Colorado, 12700 E 19th Avenue, Mailstop B112, Research Complex 2, Room 7124, Aurora, CO 80045, USA; 7 Department of Anesthesiology, Intensive Care and Pain Medicine, Johann Wolfgang Goethe-Universität Frankfurt/Main, Theodor Stern Kai 7, 60590 Frankfurt, Germany; 8 Department of Anesthesiology and Intensive Care Medicine, Eberhard Karls Universität -Tübingen, Hoppe Seyler Strasse 3, 72076 Tübingen, Germany

**Keywords:** aortic atheroma, intraaortic balloon pump, kidney injury

## Abstract

**Introduction:**

Acute kidney injury (AKI) after cardiac surgery increases length of hospital stay and in-hospital mortality. A significant number of patients undergoing cardiac surgical procedures require perioperative intra-aortic balloon pump (IABP) support. Use of an IABP has been linked to an increased incidence of perioperative renal dysfunction and death. This might be due to dislodgement of atherosclerotic material in the descending thoracic aorta (DTA). Therefore, we retrospectively studied the correlation between DTA atheroma, AKI and in-hospital mortality.

**Methods:**

A total of 454 patients were retrospectively matched to one of four groups: -IABP/-DTA atheroma, +IABP/-DTA atheroma, -IABP/+DTA atheroma, +IABP/+DTA atheroma. Patients were then matched according to presence/absence of DTA atheroma, presence/absence of IABP, performed surgical procedure, age, gender and left ventricular ejection fraction (LVEF). DTA atheroma was assessed through standard transesophageal echocardiography (TEE) imaging studies of the descending thoracic aorta.

**Results:**

Basic patient characteristics, except for age and gender, did not differ between groups. Perioperative AKI in patients with -DTA atheroma/+IABP was 5.1% versus 1.7% in patients with -DTA atheroma/-IABP. In patients with +DTA atheroma/+IABP the incidence of AKI was 12.6% versus 5.1% in patients with +DTA atheroma/-IABP. In-hospital mortality in patients with +DTA atheroma/-IABP was 3.4% versus 8.4% with +DTA atheroma/+IABP. In patients with +DTA atheroma/+IABP in hospital mortality was 20.2% versus 6.4% with +DTA atheroma/-IABP. Multivariate logistic regression identified DTA atheroma > 1 mm (*P *= *0.002, odds ratio (OR) = 4.13, confidence interval (CI) = 1.66 to 10.30), as well as IABP support (P = *0.015, OR = 3.04, CI = 1.24 to 7.45) as independent predictors of perioperative AKI and increased in-hospital mortality. DTA atheroma in conjunction with IABP significantly increased the risk of developing acute kidney injury (*P *= 0.0016) and in-hospital mortality (*P *= 0.0001) when compared to control subjects without IABP and without DTA atheroma.

**Conclusions:**

Perioperative IABP and DTA atheroma are independent predictors of perioperative AKI and in-hospital mortality. Whether adding an IABP in patients with severe DTA calcification increases their risk of developing AKI and mortality postoperatively cannot be clearly answered in this study. Nevertheless, when IABP and DTA are combined, patients are more likely to develop AKI and to die postoperatively in comparison to patients without IABP and DTA atheroma.

## Introduction

Acute kidney injury (AKI) represents one of the most detrimental complications after open heart surgery. The incidence of perioperative AKI has been reported to vary from 1% to 30%. Patients developing AKI are at higher risk for an extended hospital stay and for increased in-hospital mortality [1-3]. Several preoperative risk factors for AKI are known including medical conditions such as diabetes mellitus, congestive heart failure (CHF) or increased age. Patients with CHF, acute coronary syndrome (ACS) or inability to be weaned off cardiopulmonary bypass (CPB) are often treated with intra-aortic balloon counterpulsation (IABP). IABP support has previously been linked to the development of postoperative renal dysfunction [4-10].

The etiology of AKI following cardiopulmonary bypass and during IABP support remains poorly understood. In rare cases the development of AKI during IABP support can be explained by displacement of the IABP and subsequent occlusion of the renal arteries. To date, it remains controversial if a calcified descending thoracic aorta (DTA) could increase the incidence of AKI in patients with IABP through possible dislodgement of embolic material. However, IABP support as well as DTA atheroma has been linked independently to the development of postoperative AKI [4-10]. A displacement of aortic microemboli triggered by mechanical forces applied to the aortic wall during the use of IABP counterpulsation with subsequent impairment of the renal circulation seems possible. Intraoperative transesophageal echocardiography (TEE) is an excellent tool to monitor for the presence of aortic atheroma during cardiac surgery [11-14]. TEE is also readily available in most medical centers and is helpful for the identification of descending aortic atheroma.

To date, an association between the occurrence of AKI with the combination of DTA atheroma and/or the use of an IABP has not been well investigated. Given the importance of this for the length of in-hospital stay and in-hospital mortality we hypothesized that IABP counterpulsation in patients with DTA would lead to an increased incidence of perioperative AKI and in-hospital mortality following cardiac surgery.

In this study we confirm that DTA atheroma and IABP independently increase the risk for acute kidney injury and in-hospital mortality. We also report that the combination of DTA atheroma and IABP further increases the incidence of acute kidney injury and in-hospital mortality.

## Materials and methods

### Study design and matching

Approval for this retrospective case control study was obtained from the Institutional Review Board (IRB) of the Brigham and Women's Hospital/Harvard Medical School. Written informed consent was waived for this retrospective study.

A total of 454 patients, 50 years old and older (mean ± 68 years) with normal renal function preoperatively (creatinine < 1.5 mg/dL), who had undergone heart surgery at Brigham and Women's Hospital between January 1995 and December 2005 were retrospectively evaluated. Out of those patients we selected all patients who had received an IABP within 24 hours prior to or during surgery. Patients with preoperative renal insufficiency (creatinine ≥1.5 mg/dl) and TEE imaging-windows which were inadequate to demonstrate the severity of DTA atheroma, as well as patients who received IABP support after leaving the operating room, were excluded from the study population. Matching for the remaining 454 patients was then attempted according to the degree of DTA atheroma, performed surgical procedure, age, gender and left ventricular ejection fraction (LVEF) to one of four groups: -IABP/-DTA atheroma, +IABP/-DTA atheroma, -IABP/+DTA atheroma, +IABP/+DTA atheroma. Patients with DTA atheroma were slightly older and less likely to be male.

### Definition of preoperative medical co-morbidities

Demographics and historical data were obtained from the computerized institutional database and included age, gender, history of hypertension necessitating treatment with antihypertensive medications, diabetes mellitus, congestive heart failure, hypercholesterolemia, medications including Angiotensin-converting ezyme (ACE) -inhibitors, Aspirin (ASA) and 3-hydroxy-3-methyl-glutaryl (HMG) -CoA reductase inhibitors and use of aminocaprioc acid or aprotinin during surgery. Preoperative co-morbidities were defined according to the Society of Thoracic Surgeons (STS) standard definitions. Congestive heart failure (CHF) was defined as either paroxysmal nocturnal dyspnea, dyspnea on exertion due to CHF, a chest x-ray showing pulmonary congestion or pedal edema and dyspnea and receiving diuretics or digoxin within two weeks prior to the surgical procedure. Use of ASA, ACE-inhibitors or statins was defined as whether the patient received the medication within 24 hours prior to surgery. Diabetes mellitus indicated whether the patient had a history of diabetes, regardless of duration or need for anti-diabetic agents. Dyslipidemia was referred to as hypercholesterolemia. Criteria included the documentation of total cholesterol > 200 mg/dl or LDL > 130 mg/dl or HDL < 30 mg/dl or admission cholesterol > 200 mg/dl or triglycerides > 150 mg/dl. The criteria for hypertension included a documented history of hypertension diagnosed and treated with medication/diet and/or exercise, blood pressure > 140 systolic or > 90 diastolic on at least two occasions or currently on antihypertensive medication.

### Definition of perioperative acute kidney injury (AKI) and in-hospital mortality

Perioperative AKI was defined according to the criteria of The Society of Thoracic Surgeons (STS) as an increase in creatinine > 2.0 (mg/dl), a 100% increase from the most recent preoperative creatinine level, or need for dialysis [15]. In-hospital mortality was defined as death occurring during the hospitalization in which the original cardiac surgical intervention was performed, according to STS standards [15].

### Identification of DTA atheroma

The institutional intraoperative TEE database was used to identify cardiac surgical patients in whom imaging windows of the descending thoracic aorta were obtained. Off-line analysis of the recorded study was performed, and the degree of DTA atheroma was independently assessed by two attending cardiac anesthesiologists certified in perioperative TEE. Data from the thoracic aorta were extracted from the comprehensive TEE examination that is performed as part of routine care in applicable cardiac surgery patients. The following views were reviewed in accordance with the published Society of Cardiovascular Anesthesiologists/American Society of Echocardiography guidelines:

Descending Aorta:

1. Midesophageal descending aorta short axis at 0 degrees

2. Midesophageal descending aorta short axis at 90 degrees.

The severity of DTA atheroma was graded according to the practice guidelines for perioperative transesophageal echocardiography. A report by the American Society of Anesthesiologists and the Society of Cardiovascular Anesthesiologists Task Force on Transesophageal Echocardiography defines the grades as follows: [16] Grade 1 = no calcification, Grade 2 = intimal thickening ≤1 mm, Grade 3 = calcific protrusion 1 to 5 mm into the aortic lumen, Grade 4 = calcific protrusion > 5 mm into aortic lumen, Grade 5 = mobile atheroma. For this study, we defined significant DTA atheroma as the presence of an intraluminal plaque > 1 mm (that is, Grades 3 to 5 as described above) [17].

### Statistical analysis

Descriptive statistics were obtained on patient characteristics including demographic data, past medical history and in-hospital data. For bivariate analysis the Fishers exact test for the categorical variables and the non-parametric Mann-Whitney U test for the continuous variables were employed. Multivariate analysis was performed using logistic regression. Covariates adjusted for included all the variables that had a *P *^3^0.2 or that were felt to be clinically significant confounders. The Wald statistic was used to define the significance of the regression coefficients. A backward stepwise (likelihood ratio) technique was used for the multivariate analysis. The OR, *P *values and 95% CI were obtained from this. A *P *value of 0.05 was used to reject the null hypothesis (that the regression coefficient equates to zero). The statistical analysis was performed using JMP (Cary, NC, USA) and SPSS (Chicago, IL, USA).

## Results

### Patient characteristics and surgical procedures

Demographic data did not differ significantly between groups (Table 1) except for patients in the +DTA group who were slightly older and less likely to be men. Diabetes mellitus was more prevalent in the patient groups with calcification of the DTA, but this difference was not statistically significant. The use of preoperative drug therapy was not significantly different between groups (Table 1). Surgical procedures performed were not statistically different between groups (Table 2). The use of antifibrinolytics such as amicar (ε-aminocaproic acid) or aprotinin did not differ between groups and was not an independent predictor of AKI or death in our patient population (Table 2).

**Table 1:** Demographic data, preoperative co-morbidities and medications stratified by the presence of DTA atheroma and IABP support.

Group	-DTA/-IABP (Number = 114)	-DTA/+IABP (Number = 114)	+DTA/-IABP (Number = 113)	+DTA/+IABP (Number = 113)
Age in years (Mean ± SD)	65/± 13.7	65/± 11.8	71/± 11.2	71.4/± 9.7
Male	72 (63.0%)	66 (57.6%)	61 (53.9%)	64 (56.6%)
Female	42 (36.0%)	48 (42.4%)	52 (46.0%)	49 (42.9%)
HTN	74 (62.2%)	81 (68.6%)	75 (70.8%)	87 (79.8%)
DM	32 (26.9%)	31 (26.3%)	47 (44.3%)	46 (42.2%)
HCL.	80 (67.2%)	83 (70.3%)	78 (73.6%)	82 (75.2%)
ACE	74 (62.2%)	50 (42.3%)	63 (59.4%)	64 (58.7%)
ASA	86 (72.3%)	76 (64.4%)	77 (72.6%)	86 (78.9%)
Statins	76 (63.9%)	67 (56.8%)	68 (64.1%)	67 (61.5%)
LVEF (%)	41.4/± 15.6	43.0/± 15.3	39.6/± 15.4	38.6/± 15.1
CHF	41 (34.5%)	57 (48.3%)	45 (42.5%)	64 (58.7%)

ACE, angiotension converting enzyme inhibitors; ASA, aspirin; CHF, congestive heart failure; DM, diabetes mellitus; HCL, hypercholesterolemia; HTN, hypertension; LVEF, left ventricular ejection fraction; Statins, HMGCoA reductase inhibitors.

**Table 2:** Intraoperative variables and anti-fibrinolytics stratified by the presence of DTA atheroma and IABP support.

Group	-DTA/-IABP (Number = 114)	-DTA/+IABP (Number = 114)	+DTA/-IABP (Number = 113)	+DTA/+IABP (Number = 113)
CABG^a^	67 (58.70%)	68 (59.6%)	59 (52.2%)	60 (53.1%)
Valve^b^	14 (12.2%)	16 (14.0%)	14 (12.4%)	13 (11.5%)
CABG and Valve^c^	29 (25.4%)	26 (22.8%)	37 (32.7%)	37 (32.7%)
Others^d^	4 (3.5%)	4 (3.5%)	3 (2.6%)	3 (2.7%)
CPB time in min (Mean ± SD)	137 ± 99.5	142 ± 73.6	150.2 ± 78.4	174.8 ± 82.1
CX time in min (Mean ± SD)	95.9 ± 45.9	91.8 ± 44.7	109 ± 56.7	110.9 ± 55.6
Amicar	96 (84.2%)	93 (81.5%)	91 (80.5%)	93 (82.3%)
Aprotinin	18 (15.7%)	21 (18.4%)	22 (19.5%)	20 (17.7%)

^a^includes primary and reoperation; ^b, c^primary, ReOp and double valves; ^d^LVA repair, heart transplant, TMLR, Ascending Aorta repair, VSD repair.

CABG, coronary artery bypass graft; CPB time, cardio-pulmonary bypass time; CX time, cross-clamp time; SD, standard deviation

### AKI and in-hospital mortality

**Figure 1:**
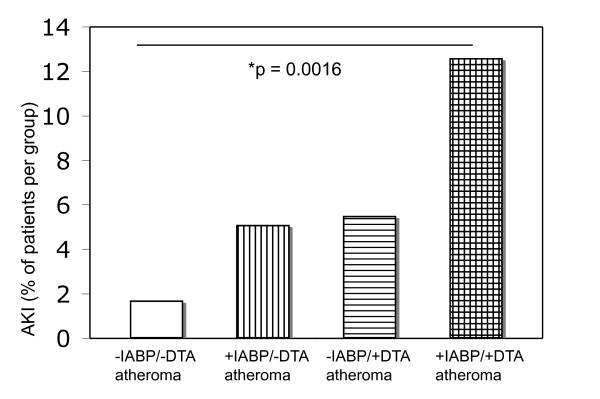
**Incidence of perioperative acute kidney injury (AKI) in the presented study population**. DTA, descending thoracic aortic atherosclerosis; IABP, intraaortic balloon counterpulsation.

The total incidence of AKI was 6.4% within the study population. The analysis showed that 3.9% of the patients without IABP support developed AKI compared with 8.8% of the patients with IABP. The incidence and the ORs of AKI within groups were as follows: -DTA atheroma/-IABP: 1.7%, -DTA atheroma/+IABP: 5.1%, +DTA atheroma/-IABP: 5.5%, and +DTA atheroma/+IABP: 12.6%. Using Fisher's exact test, patients with the combination of IABP and DTA atheroma (+IABP/+DTA atheroma) had a significantly increased risk of developing postoperative AKI (*P *= 0.0016, relative risk increase 7.062, CI = 1.64 to 30.4) when compared to patients without IABP and without DTA atheroma (-IABP/-DTA atheroma). Patients with either IABP or DTA atheroma showed an increase in AKI postoperatively, which was not statistically significant when compared to either group (Figure 1).

In the univariate analysis, patients who developed postoperative AKI were more likely to be older than 65 years (*P *= 0.008); have a history of CHF (*P *= 0.026), undergoing a valve procedure (*P *= 0.012) and have DTA calcification (*P *= 0.003) or an IABP (*P *= 0.002) (Table 3). Multivariate logistic regression controlling for confounders such as patient demographics, preoperative risk factors, medications and intraoperative variables demonstrated a history of CHF (*P *= 0.026, OR = 2.81, CI = 1.13 to 6.97), the use of IABP (*P *= 0.015, OR = 3.04, CI = 1.24 to 7.45) and DTA calcification (*P *= 0.002, OR = 4.13, CI = 1.66 to 10.30) as independent predictors for the development of AKI in our patient population (Table 4). An analysis of effects (the chi-square test statistics and associated *P*-values) indicates that IABP and calcification are significantly associated with increased postoperative AKI individually, but not the interaction between the two.

**Table 3:** Univariate analysis stratifying the risk for acute kidney injury (AKI) and in-hospital mortality (Number = 454).

	AKI (*P*-value)	Mortality (*P*-value)
DTA calcification	0.003*	0.04*
IABP	0.002*	0.036*
LV dysfunction	0.37	0.179
ACE inhibitors	0.88	1.0
ASA	0.63	0.53
Statin	0.23	0.34
Age (> than 65 years)	0.008*	0.04*
DM	0.07	1.0
HTN	0.10	0.40
CHF*	0.026*	0.01*
Cardiogenic shock	0.13	0.07
CX time	0.20	0.024*
CPB time	0.11	0.001*
Aprotinin	0.04	0.83
Aminocaproic Acid	0.04	0.83

* statistically significant (*P *< 0.05)

ACE, angiotension converting enzyme inhibitors; ASA, aspirin; CHF, congestive heart failure; CX time, cross-clamp time; DM, diabetes mellitus; DTA, descending thoracic aorta; HTN, hypertension; IABP, intra-aortic balloon pump; LV dysfunction, left ventricular dysfunction; LVEF, left ventricular ejection fraction; Statins, HMGCoA reuctase inhibitors.

**Table 4:** Independent predictors of postoperative Acute Kidney Injury (AKI) by multivariate analysis (Number = 454).

	*P*-value	OR	95% CI
DTA calcification	*0.002	4.13	1.66-10.30
IABP	*0.015	3.04	1.24-7.45
CHF	*0.026	2.81	1.13-6.97

CHF, congestive heart failure; CI, confidence interval; DTA, descending thoracic aorta; IABP, intra-aortic balloon pump; OR, odds ratio.

The incidence of in-hospital mortality among all patients was 11.3% and included 6.6% of patients without an IABP versus 16.2% of patients with IABP support. The incidence of in-hospital mortality by Group was: -DTA atheroma/-IABP: 3.4%, -DTA atheroma/+IABP: 8.5%, +DTA atheroma/-IABP: 6.4%, and +DTA atheroma/+IABP: 20.2%.

**Figure 2:**
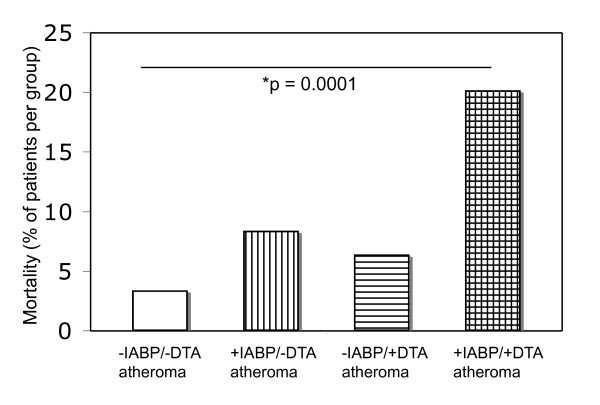
**Incidence of perioperative mortality in the presented study population**. DTA, descending thoracic aortic atherosclerosis; IABP, intraaortic balloon counterpulsation.

Using Fisher's exact test, patients with the combination of IABP and DTA atheroma (+IABP/+DTA atheroma) had a significantly increased risk of increased postoperative mortality (*P *= 0.0001, relative risk increase 5.7, CI = 2.04 to 15.9) when compared to patients without IABP and without DTA atheroma (-IABP/-DTA atheroma) (Figure 2).

In the univariate analysis, patients who died were more likely to be older than 65 years (*P *= 0.008), have a history of CHF (*P *= 0.01), receiving either aminocaproic acid (*P *= 0.04) or aprotinin (*P *= 0.041) and have DTA calcification (*P *= 0.04) or an IABP (*P *= 0.036) (Table 3). Multivariate logistic regression controlling for confounders such as patient demographics, preoperative risk factors, medications and intraoperative variables showed age (*P *= 0.015, OR = 3.53, CI = 1.28 to 9.75), perfusion time (*P *= 0.002, OR = 1.01, CI = 1.00 to 1.01), the use of an IABP (*P *= 0.016, OR = 2.61, CI = 1.19 to 5.70) and DTA calcification (*P *= 0.012, OR = 2.69, CI = 1.24 to 5.83) to be independent risk factors for mortality (Table 5).

**Table 5:** Independent predictors of postoperative Mortality by multivariable analysis (Number = 454).

	*P*-value	OR	95% CI
DTA calcification	*0.012	2.69	1.24-5.83
IABP	*0.016	2.61	1.19-5.70
Age	*0.015	3.53	1.28-9.75
Perfusion time	* < 0.002	1.01	1.00-1.01

CI, confidence interval; DTA, descending thoracic aorta; IABP, intra-aortic balloon pump; OR, odds ratio.

An analysis of effects (the chi-square test statistics and associated *P*-values) indicates that IABP and calcification are significantly associated with in-hospital mortality individually, but not the interaction between the two.

## Discussion

Postoperative AKI remains a severe complication following cardiac surgery, and is associated with a prolonged hospital stay and an increased risk for in-hospital mortality [18-20]. The etiology of postoperative AKI has been attributed to embolic events, inflammation, ischemia-reperfusion injury, intravenous contrast and a variety of medications [20,21]. In addition, the use of an IABP has been associated with an increase in the risk of perioperative AKI [4-7]. The potential benefit of using an IABP in providing perioperative circulatory support must be weighed against the potentially lethal complication of AKI in critically ill patients [22-24]. We now confirm that DTA atheroma is a risk factor for the development of perioperative AKI in cardiac surgery patients. Moreover, we also show that the combined presence of DTA atheroma and IABP significantly increases the risk of perioperative AKI in-hospital mortality when compared to control subjects without IABP and without significant DTA atheroma. Adding an IABP to a patient population with already severe DTA atheroma did not increase the overall risk for AKI and/or mortality significantly.

Aortic atheroma has been previously linked to postoperative renal injury. MacKensen *et al. *showed that ascending aortic atheroma is an independent risk factor for postoperative renal failure [21]. In their study, descending aortic atheroma alone did not demonstrate an increased risk for postoperative renal injury. In addition, atheroma of the aorta has also been associated with an increased incidence of perioperative adverse outcomes including stroke, multiorgan failure and death [25,26]. The degree, mobility and location of aortic atheroma are associated with an increased risk for systemic embolization and cerebral stroke [11-14]. Intraoperative TEE has become the standard imaging modality for identifying aortic atheroma and influencing therapy in 8% to 17% of cases [27]. Katz *et al. *showed that aortic arch artheroma, assessed intraoperatively with TEE, was the only variable to predict cerebral embolic events after cardiac surgery [17,27]. However, prior studies evaluating the risk of AKI in patients undergoing surgery with and without the use of an IABP have not included an evaluation and measurement of DTA atheroma severity.

The exact mechanism of IABP-induced aortic damage remains unclear. Tierney *et al. *demonstrated that the use of an IABP can cause atheromatous lesions in the aorta to rupture and cause fissures, even in the non-atheromatous vessel [28], which is in keeping with our initial hypothesis that dislodged atheromatous material can lead to downward stream embolization of renal vessels when using IABP support. Ramnarine *et al. *demonstrated that the use of IABP was an independent risk factor for both AKI and death in a heterogeneous population (isolated coronary artery bypass grafting (CAGB) and valve procedures, as well as combined CABG and valve procedures) of more than 7,600 patients [6]. Those patients who required IABP in this study were generally older, had preoperative renal dysfunction and severely decreased LV function. In a large prospective study of over 43,000 cardiac surgical patients Chertow *et al. *found that the preoperative use of IABP was an independent risk factor for postoperative AKI (OR: 4.57; 95% CI 3.57 to 5.86) [20]. However, the overall incidence of AKI in this patient population was only 1.1%, since only patients who required dialysis were included in the analysis of postoperative AKI. In another study of 2,672 patients undergoing CABG, 7.9% of patients developed AKI and 0.7% of patients required postoperative dialysis [8]. Recently, Wijeysundera *et al. *also demonstrated that the pre-operative placement of an IABP significantly increased the risk for AKI in a heterogeneous group of cardiac surgical patients [29]. None of these previous investigations included DTA atheroma as a variable in the analysis of perioperative morbidity and mortality. Fabbian *et al. *were able to link the degree of aortic calcification with renal failure. In their retrospective analysis, they assessed the presence of aortic arch calcification examined by chest radiography and the incidence of dialysis-dependent chronic renal failure [30]. Davila-Roman *et al. *could link ascending aortic artherosclerosis with the development of acute renal injury after CABG surgery [10]. In their prospective study they investigated 978 patients and used epiaortic echocardiography of the ascending aorta as a surrogate marker for DTA atheroma.

While our study provides novel insight into the influence of DTA atheroma and IABP on the incidence of AKI and mortality following cardiac surgery, certain limitations are worth noting including the use of a retrospective evaluation and a relatively small number of patients. While a prospective study involving a randomized assignment to the use of an IABP in a large, multicenter investigation might be ideal, the ethical limitations and purported clinical benefit of IABP in high risk populations would significantly restrict its feasibility. Furthermore, in our population, patients with significant DTA atheroma were also more likely to be older and have lower LVEF with CHF. We attempted to statistically control for these potential confounders, which ultimately allowed us to demonstrate significant associations between DTA and IABP with AKI. We also acknowledge that our assessment of renal injury using postoperative peak serum creatinine levels as the only marker is controversial and not perfect; however, it is commonly used in the clinical setting. The addition of creatinine clearance data or novel biomarkers such as NGAL might have been beneficial, but could not be obtained due to the retrospective nature of this study [31].

## Conclusions

In summary, the results of our study highlight the importance of the independent and interdependent influence of DTA atheroma and IABP support on perioperative AKI and mortality following cardiac surgery. Unfortunately, we were not able to show a clear independent association that the combination of IABP and DTA atheroma causes an increase of AKI and/or in-hospital mortality in cardiac surgical patients. We were able to show though that the combination of IABP and DTA atheroma increases AKI and in-hospital mortality when compared to patients without IABP or DTA disease. Further larger studies are needed to clarify this clinically important question.

While the risk benefit ratio of IABP counterpulsation may still be favorable in patients regardless of the presence of DTA atheroma, identifying the severity and exact location of aortic atheroma, may be useful for risk stratification, directing optimal positioning of an IABP under echocardiographic guidance, or influencing the decision to utilize alternative sites for IABP placement including nonconventional transbrachial techniques [32].

## Abbreviations

ACE: angiotension converting enzyme inhibitor; ACS: acute coronary syndrome; AKI: acute kidney injury; ASA: aspirin; CABG: coronary artery bypass graft; CHF: congestive heart failure; CI: confidence interval; CPB time: cardio-pulmonary bypass time; CX time: cross-clamp time;DM: diabetes mellitus; DTA: descending thoracic aorta; HCL: hypercholesterolemia; HDL: high density lipoprotein; HTN: hypertension; HMGCoA reductase inhibitors: 3-hydroxy-3-methyl-glutaryl-CoA reductase inhibitors; IABP: intraaortic balloon counterpulsation; IRB: Institutional Review Board; LDL: low density lipoprotein; LVEF: left ventricular ejection fraction; OR: odds ratio; SD: standard deviation; STS: society of thoracic surgeons; TEE: transesophageal echocardiography.

## Competing interests

Stanton K. Shernan discloses a consultancy with Philips Healthcare, Inc. All other authors declare that they have no competing interests.

## Authors' contributions

HKE, ST, and AM designed the study and analyzed the data. JDR and PSS collected the data. GL collected and analyzed the data and designed the study. TMB collected the data and wrote and revised the manuscript. AM analyzed the data. MNM and PR collected the data, designed the study, analyzed the data, and wrote and revised the manuscript. SKS designed the study, analyzed the data, and wrote and revised the manuscript. All authors have read and approved the manuscript for publication.
